# Iris color distribution in the United States of America

**DOI:** 10.1371/journal.pone.0312913

**Published:** 2025-09-29

**Authors:** Faris Hashem, Cristina Menicacci, David B. Granet

**Affiliations:** 1 Ratner Children’s Eye Center of the Shiley Eye Institute, University of California San Diego, La Jolla, California, United States of America; 2 Division of Ophthalmology, Department of Surgery, Faculty of Medicine, University of Tabuk, Tabuk, Saudi Arabia; 3 Ophthalmology Unit, Santa Maria alle Scotte Hospital, Siena, Italy; 4 Viterbi Family Department of Ophthalmology, Shiley Eye Institute, University of California San Diego, La Jolla, California, United States of America; University of Huddersfield, UNITED KINGDOM OF GREAT BRITAIN AND NORTHERN IRELAND

## Abstract

**Objective:**

To document the distribution of iris color in the United States of America.

**Design:**

This original investigation is an epidemiologic assessment of eye color data from all 50 states’ Department of Motor Vehicles (DMV).

**Participants:**

All driver’s license holders data nationwide (age 16 years and older, both genders) were requested from the Department of Motor Vehicles (DMVs) in each state. Driver’s license holders from states whose DMV did not participate in the study due to special state-specific regulations, did not compile iris color information, or did not respond were then excluded.

**Main Outcome Measures:**

Self reported eye color information was obtained from the DMVs databases of each driver’s license applicant self-reported eye color information.

**Methods:**

All 50 states’ Department of Motor Vehicles in the USA were contacted using various methods and the database of driver licenses eye color for current active licenses (without including any personal information) was requested. Any iris color beyond grey, blue, green, hazel, or brown/black was categorized as “others”.

**Results:**

Iris color of 235,423,085 driver’s license holders (DLHs) from 31 states was collected. The data show that brown/black iris color was documented in 124,811,254 DLHs (53%), blue in 55,797,458 DLHs (23.7%), hazel in 24,152,854 DLHs (10.3%), green in 21,258,873 DLHs (9%), grey in 1,597,675 DLHs (0.7%), and other iris colors in 7,804,971 DLHs (3.3%).

**Conclusions:**

Utilizing information from over 230 million driver’s license holders, this report is the largest study of iris color distribution representing the United States of America, thus providing a valuable source for future eye disease and other sociological research. The data show that the most prominent iris color in the United States of America is brown/black, then blue, hazel, green, other iris colors, and grey.

## Introduction

The United States of America is the third-largest country in the world in terms of population and area [1,2]. With people from different origins and backgrounds, the United States presents a unique community within which to document iris pigmentation, commonly noted as “iris color” distribution.

Previously, iris color distribution has been documented in limited populations. In 1921, the Medical Department of the United States Army published the distribution of veterans’ iris colors [3]. Also, in the late 1900s, the National Center for Health Statistics (NCHS) conducted the National Health and Nutrition Examination Surveys (NHANES) I and III, from 1971 to 1974 and from 1988 to 1994, respectively, which documented iris color distribution in limited populations of the United States [4,5].

From a medical perspective, an association between different iris pigmentation/color and eye diseases, such as cataracts and melanomas, has been reported [6–11]. Without a database of normal distribution, understanding the role of iris color in disease or relating to cultural impact is not possible. If bias towards a certain iris color exists, whether in ocular disease or sociologic interactions, a control group would be needed for comparison.

This study identifies the distribution of iris color in the United States of America. The Department of Motor Vehicles were utilized as they maintain demographic information on drivers in each state, representing a high percentage of the U.S. population.

## Materials and methods

This study analyzed publicly available data obtained from state Departments of Motor Vehicles. All data were de-identified and contained no personal health information (PHI) or personally identifiable information. In accordance with institutional guidelines, IRB approval was not required for research involving publicly available, non-identifiable datasets. The Department of Motor Vehicle (DMV) in each of the 50 States was contacted. In some states these have different names, such as the Department of Transportation, Department of Driver Services, or Department of Public Safety.

The governmental agencies were contacted via email, electronic request, mail, or phone, depending on the listed contact information through each official website. In addition, some states required Freedom of Information Act (FOIA) requests, state-specific request forms, and an official letter from the initiating institute. Fees for programming and reporting varied from $10 to $600. The database of driver licenses broken down by iris color for current and active driver’s licenses without any further personal information was requested in order to get cooperation. The self-reported iris colors were classified into grey, blue, green, hazel, and brown/black. Any different iris color given (including pink, ruby, amber, maroon, dichromatic, and unknown) was considered as “others”.

## Results

After contacting the 50 state agencies that maintained driver’s license data, replies from 42 DMVs were received. The remaining eight DMVs were contacted multiple times utilizing all available contact information with no response: Montana, Nevada, New Mexico, Hawaii, Iowa, Louisiana, Mississippi, and Rhode Island. Five states said they do not compile iris color information for their driver’s license issuance: Maryland, North Dakota, Massachusetts, South Carolina, and Florida. Due to state-specific regulations, five DMVs were not able to provide the requested data; Arkansas and Tennessee have a state law that prevents the release of such information to an out-of-state citizen, Alabama’s DMV Legal Unit did not approve the release of data, New Jersey’s Open Public Records Act (OPRA) OPRA does not require the creation of a new government record such as an iris color data request, Colorado’s DMV replied that they do not have a report containing this data. Oklahoma’s Department of Public Safety responded that “no responsive records have been identified to date”, even though this state’s driver’s licenses have eye color information. Thus, of the 42 states that responded, 31 have provided the requested data.

The combined data from these 31 states totaled 235,423,085 unique licenses.

Using this data, the overall iris color distribution in the United States of America is as follows: brown/black, 53%; blue, 23.7%; hazel, 10.3%; green, 9%; grey, 0.7%; and other iris colors, 3.3%.

**Fig 1:**
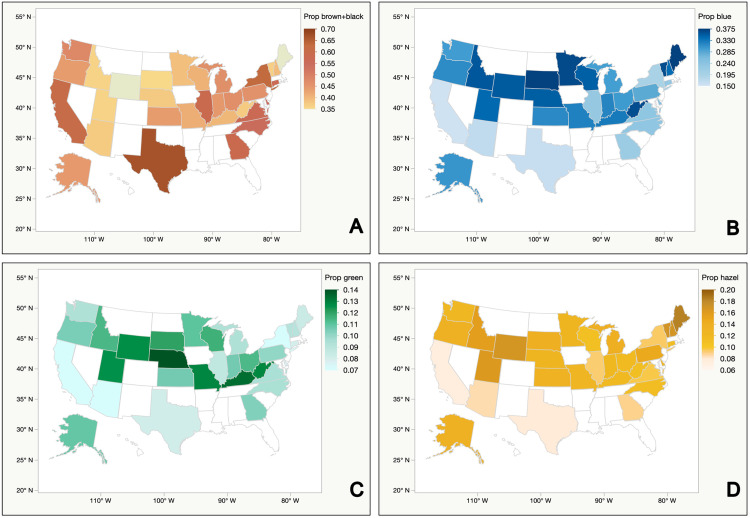
U.S. maps showing the prevalence of different iris colors in the 31 participating states according to the self-reported DMV data of active and current driver’s licenses as of 2016. A: prevalence of brown/black iris color; B: prevalence of blue iris color; C: prevalence of green iris color; D: prevalence of hazel iris color.

Most states demonstrated the same iris color distribution: brown/black > blue > hazel > green > grey. (Fig 1 and Table 1).

**Table 1:** The prevalence of different iris colors in the 31 participating states according to the self-reported DMV data of active and current driver’s licenses in 2016.

	Grey		Blue		Green		Hazel		Brown/Black		Other^a^	
State	N	%	N	%	N	%	N	%	N	%	N	%
Alaska	3,462	0.6	167,843	30.5	59,948	10.9	74,969	13.6	243,956	44.3	0	0.0
Arizona	40,060	0.4	1,901,827	17.1	783,991	7.0	944,949	8.5	4,173,370	37.5	3,285,198	29.5
California	293,567	0.6	7,826,958	15.3	3,572,827	7.0	3,840,904	7.5	31,254,208	61.0	4,410,395	8.6
Connecticut	9,127	0.5	399,530	22.8	138,707	7.9	197,146	11.3	995,969	57.0	8,240	0.5
Delaware	3,828	0.5	176,063	25.1	54,882	7.8	88,599	12.6	378,773	53.9	95	0.0
Georgia	44,450	0.6	1,597,350	21.6	768,315	10.4	661,140	8.9	4,332,961	58.5	1,577	0.0
Idaho	16,799	0.8	729,043	35.8	232,892	11.4	324,463	15.9	734,957	36.1	0	0.0
Illinois	109,393	0.6	3,808,559	22.6	1,469,440	8.7	1,555,882	9.2	9,886,543	58.7	0	0.0
Indiana	74,192	0.9	2,498,654	32.0	832,098	10.7	928,466	11.9	3,479,266	44.5	65	0.0
Kansas	23,525	0.8	957,326	31.6	322,978	10.6	404,549	13.3	1,324,318	43.7	0	0.0
Kentucky	38,856	0.8	1,513,838	32.9	616,790	13.4	545,383	11.9	1,834,356	39.9	48,831	1.1
Maine	9,969	0.5	742,942	37.7	164,298	8.3	363,181	18.5	687,448	34.9	458	0.0
Michigan	41,791	0.5	2,664,507	29.6	842,956	9.4	1,274,948	14.2	4,182,736	46.4	0	0.0
Minnesota	46,900	0.7	2,444,702	36.8	731,097	11.0	824,910	12.4	2,589,096	39.0	6,561	0.1
Missouri	34,131	0.7	1,524,377	32.3	606,478	12.9	601,219	12.8	1,949,182	41.3	0	0.0
Nebraska	6,777	0.5	465,723	35.3	185,147	14.0	164,409	12.5	495,788	37.6	201	0.0
New Hampshire	7,081	0.5	482,573	34.1	128,358	9.1	238,852	16.9	557,031	39.4	230	0.0
New York	107,276	0.7	2,999,476	19.6	1,054,923	6.9	1,434,430	9.4	9,686,619	63.4	0	0.0
North Carolina	10,910	0.4	652,973	23.5	263,952	9.5	311,591	11.2	1,539,624	55.4	862	0.0
Ohio	78,651	0.6	3,953,518	30.0	1,497,369	11.4	1,522,701	11.6	6,118,970	46.5	0	0.0
Oregon	36,583	0.7	1,684,784	31.4	564,281	10.5	731,600	13.6	2,344,131	43.7	2,169	0.0
Pennsylvania	68,531	0.8	2,509,951	28.4	888,370	10.1	1,303,562	14.8	4,055,579	45.9	5,782	0.1
South Dakota	4,152	0.6	271,909	38.0	86,156	12.0	101,231	14.2	251,754	35.2	76	0.0
Texas	162,235	0.6	4,098,085	16.2	2,056,065	8.1	2,027,419	8.0	17,003,394	67.1	1,672	0.0
Utah	10,210	0.5	672,564	34.4	246,285	12.6	320,213	16.4	704,663	36.1	429	0.0
Vermont	5,987	0.8	287,663	36.5	71,761	9.1	133,416	16.9	289,419	36.7	0	0.0
Virginia	59,655	0.7	1,972,492	23.6	793,401	9.5	808,741	9.7	4,734,024	56.6	0	0.0
Washington	102,578	1.1	2,906,481	29.8	911,797	9.4	1,167,812	12.0	4,655,370	47.8	1,928	0.0
West Virginia	48,983	2.2	796,268	36.5	280,181	12.9	240,792	11.0	813,046	37.3	0	0.0
Wisconsin	92,273	1.2	2,760,143	35.1	915,839	11.7	860,465	11.0	3,198,070	40.7	30,202	0.4
Wyoming	5,743	0.6	329,336	35.6	117,291	12.7	154,912	16.8	316,633	34.3	0	0.0
**Total** ^**b**^	1,597,675	0.7	55,797,458	23.7	21,258,873	9.0	24,152,854	10.3	124,811,254	53.0	7,804,971	3.3

^a^Other iris color group consists of pink, maroon, dichromatic, and unknown eye colors.

^b^Total for the 31 participating states. N: The total number of populations with each iris color. %: The percentages of people with each iris color from the total population of the participating states.

## Discussion

In many ways, the iris color represents a region of origin and, in others, ethnicity despite its inheritance not being considered in a simple Mendelian fashion. If iris color impacts ocular disease, then knowing the normal distribution of that color is needed for comparison. Similarly, if there is a systematic bias regarding iris color in hiring practices, promotion, elections, choice of actors, or dating partners, a comparison to a normal distribution would be needed.

Previous attempts at identifying iris color distribution have been flawed by limited group inclusion and size or poor sampling [3–5]. The current report attempts to get a broad population sample in one of the most heterogeneous countries globally. The distribution of iris color, brown/black>> blue>> hazel>> green>> gray, reflects the migration patterns to the USA, which have likely changed and will change over time. Internationally, some more homogeneous regions like Scandinavia or Asia might differing iris color distributions.

Interestingly, three states showed blue iris color as the most prominent, including Maine, Wyoming, and South Dakota. Seven states demonstrated green iris color more commonly than hazel (within the overall order of color): Missouri, Texas, West Virginia, Wisconsin, Georgia, Kentucky, and Nebraska. These disparities might be explained by the immigration history of the United States, in which Europeans migrated to the Northwest and Midwest states. On the other hand, Asians migrated to the Pacific region [12–14]. It is also possible that a self-reporting bias leads to green being chosen over hazel in these states.

The United States Census Bureau data shows that in 2016, the total population of people 16 years and older in the country was (257,955,453), 78% of them are Whites, 13% are African-Americans, 6% Asians, 1% American-Indians, < 1% Hawaiians, and 2% of more than one “mixed” race. People who identify their origin as Spanish, Hispanic, or Latino are related to a region of origin and may be of any race. Thus, the percentage of Hispanics would not be added to percentages for racial categories.

When comparing the U.S. census race data to the collected iris color data, it was interesting to find that the three states that showed blue color more prominent also have different race distribution and order: Maine, Wyoming, and South Dakota showed that White race was noticeably larger than the U.S. distribution (96%, 94%, 88%, respectively). Additionally, the African-American race was much less than the U.S. distribution (ranging between 1–2%). This may explain the dominance of blue iris color in these states.

Iris colors could be an essential determinant in identifying populations at risk of certain ocular diseases. Published clinical research examined the association between iris color and ocular diseases and their possible role as risk factors. A meta-analysis published in 2014 concluded that cataracts were associated with darker iris colors and found no evidence of an increased risk of age-related macular degeneration (AMD) with a particular iris color [7]. A different meta-analysis studied the potential risk factors for AMD, and their findings suggest that having darker iris colors is protective, but this relation was not statistically significant [15]. Also, other reports found that AMD is related to lighter iris colors [16–18]. As for glaucoma, one study found that high intra-ocular pressure, a strong determinant for glaucoma, is more common in African Americans with dark iris colors [19].

These reports found a possible relationship between iris color and some ocular diseases, but were conducted on limited populations. Hence, the need for population-based studies to assess iris color as a potential risk factor for ocular diseases is high. Collecting iris color and ocular diseases data nationwide at an individual level will solidify the evidence of the association of iris color with ocular diseases.

While collecting the data, we encountered different means of communication with several intended governmental entities, including variations in nomenclature. In following up with all the “DMVs” that responded, different concerns arose, like requesting specific forms and documents and variable time frames for delivering the information request. The distinctive laws of each state explain the indicated differences. It is possible that the idiosyncratic approaches of each state affected the results in the methods used to collect and report data.

Although about three-fifths of the intended states (or about two-thirds of the given population) responded, it is still possible there is an unintended systematic bias in the results. The states without data or non-responsive do not seem prima facie to represent a specific and different cohort of iris color. A self-selection bias can be caused by the different needs for obtaining a driver’s license from one place to another, depending on public transportation and other reasons. Thus, the factors mentioned above may cause selection bias. Additionally, people migrate from one state to another and obtain new driver’s licenses from the new state, which may lead to duplication of data and a possible migration bias, affecting the study results. The self-reported nature of iris color may lead to misclassification bias.

Interestingly, iris color distribution likely reflects immigration, migration, and other epidemiologic patterns from marriage to birth rates. As iris color data is hard to identify, tracking these changes over time is difficult. Further, a correlation to those larger epidemiologic patterns is outside the scope of this report.

It would be valuable if all DMVs nationwide used a standardized approach to information on driver’s licenses. If iris color data were entered by a DMV representative at the time of visual acuity testing using a standardized scale, more robust data could be obtained. The availability of such a database is important for future research linked to different iris colors ranging from ocular disease to sociologic impact.

The novelty of this current study is shown in documenting the prevalence of iris color in the United States. This study is valuable for future research assessing the relationship between certain iris colors, ocular diseases, and other diseases and health issues. Additionally, this report will be valuable in evaluating if there is a systematic bias regarding iris color in hiring practices, promotion, elections, choice of actors, dating partners, and more.

To the author’s knowledge, this is the largest documented iris color distribution database and represents the United States of America population. Given the over 230 million data points, the likelihood of this database being valid is high, with the chance of systematic or significant error being low. Thus, the overall iris color distribution in the United States of America using this data can be considered: brown/black 53%; blue 23.7%; hazel 10.3%; green 9%; grey <1%; and other iris colors 3.3%.
